# Long-Term Comparative Outcomes of TNF-α Antagonists vs. Vedolizumab as First-Line Biologic Therapy for Refractory Ulcerative Proctitis: A Propensity-Matched Study

**DOI:** 10.3390/biomedicines14051135

**Published:** 2026-05-17

**Authors:** Ayushi Shah, Azhar Hussain, Ahmad Nawaz, Abdelkader Chaar, Avleen Kaur, Mark M. Aloysius, Bishnu Sapkota, Savio John, Idan Goren

**Affiliations:** 1 Division of Gastroenterology, SUNY Upstate Medical University, Syracuse, NY 13210, USA; 2 Department of Medicine, SUNY Upstate Medical University, Syracuse, NY 13210, USA

**Keywords:** ulcerative proctitis, refractory proctitis, anti-TNF, vedolizumab, 5-ASA, outcomes

## Abstract

**Background**: The optimal first-line biologic therapy for refractory ulcerative proctitis (UP) remains uncertain, largely because patients with UP are frequently excluded from biologic clinical trials, limiting evidence to guide treatment selection. This study evaluated outcomes among patients with UP treated with first-line TNF inhibitors or vedolizumab. **Methods**: We performed a retrospective cohort study using the TriNetX database between 1995 and 2023. Propensity score matching was applied to balance demographics, laboratory parameters, and baseline medications. Primary outcomes included corticosteroid use, all-cause emergency room (ER) visits and hospitalizations, and colectomy, assessed at 6, 12, and 24 months after initiation of TNF inhibitors or vedolizumab. Secondary outcomes described real-world biologic usage patterns in UP. **Results**: Among 641 patients with UP receiving advanced therapy, the most commonly used biologics were adalimumab (39%), infliximab (27%), and vedolizumab (26%). Ustekinumab was used in 12% of patients. In matched analyses, TNF inhibitor therapy was associated with reduced ER visits and hospitalizations at 6 and 12 months compared with vedolizumab (6 months: 14.3% vs. 25%, aOR 0.50, *p*-value 0.03; 12 months: 16.1% vs. 35.2%, aOR 0.35, *p*-value 0.01). By 24 months, no significant differences were observed. Corticosteroid use and colectomy rates were similar across therapies at all time points. In a subgroup comparison between adalimumab and vedolizumab, results were consistent with the primary analysis, with lower short-term ER visits and/or hospitalizations among patients receiving adalimumab. **Conclusions**: In this propensity-matched analysis, TNFi therapy was associated with lower short-term healthcare utilization, with no significant differences observed in corticosteroid use. These findings should be interpreted cautiously given nonspecific outcomes and potential residual confounding from unmeasured disease variables such as endoscopic activity.

## 1. Introduction

Ulcerative proctitis (UP), a localized form of ulcerative colitis (UC), is characterized by inflammation confined to the rectum. Topical or combined oral and topical 5-aminosalicylic acid (5-ASA) is the first-line treatment for UP.

Although UP is limited in extent, it can cause debilitating symptoms, and approximately one-third of patients experience refractory disease [1]. Poorly controlled UP is associated with a higher risk of proximal extension and an increased likelihood of colectomy [2,3]. For patients with 5-ASA-refractory disease or intolerance, escalation to advanced therapies—including biologics and small molecules—may be necessary. Achieving effective disease control is therefore essential.

Patients with UP are frequently excluded from randomized controlled trials of advanced therapies for UC, resulting in limited high-quality data and long-term outcomes to guide the optimal use of advanced therapies [1,4,5]. Topical therapies such as tacrolimus have been studied in small randomized controlled trials (RCTs) and case series, but the quality of evidence is currently low and has not gained momentum in clinical practice for refractory UP as much as the step-up to advanced therapy. Among advanced therapies, only tofacitinib and etrasimod have prospective studies demonstrating drug persistence and efficacy specifically for patients with UP [6,7]. Anti-tumor necrosis factor agents (TNFis) and vedolizumab are commonly used first-line advanced therapies in patients with UC; however, data on their efficacy in patients with UP are limited to small observational studies [1,4,5,8,9]. We aimed to evaluate the clinical outcomes of refractory UP patients treated with TNFis or vedolizumab as first-line advanced therapy.

## 2. Materials and Methods

### 2.1. Study Population

De-identified electronic medical records from 140 global healthcare organizations (HCOs) were accessed via the TriNetX database to identify adults with UP using the ICD-10 codes K51.2, K51.20, K51.21, K51.211, K51.212, K51.213, K51.214, K51.218, and K51.219 (Supplementary Table S1). To ensure the accuracy of the cohort and the exclusion of those with more extensive disease, we systematically excluded any patient with ICD-10 codes indicating more extensive forms of ulcerative colitis—including ulcerative proctosigmoiditis (K51.3), left-sided colitis (K51.5), pancolitis (K51.0), and other unspecified or extensive forms (K51.5, K51.8, K51.9). Additionally, all patients with any ICD-10 codes consistent with Crohn’s disease (K50.x) were excluded (Supplementary Table S1). We further refined the cohort by excluding patients with non-IBD immune-mediated conditions that may be treated with anti-TNF agents or systemic steroids such as rheumatoid arthritis (M05.x, M06.x), systemic lupus erythematosus (M32.x), psoriasis (L40.x), ankylosing spondylitis (M45.x), multiple sclerosis (G35), and systemic vasculitides (M30.x, M31.x, I77.6), to reduce potential treatment-related confounding.

UP patients treated with TNFis or vedolizumab as first-line advanced therapy between 1 January 1995 and 1 May 2023 were included in the analysis. These were confirmed as first-line therapies by excluding any exposure to biologics before the index event of starting a biologic, which was either TNFis or vedolizumab. Analysis was performed in January 2025.

### 2.2. Study Outcomes

The primary outcomes were corticosteroid use, all-cause ER visits and hospitalizations, and colectomy, assessed at 6, 12, and 24 months after initiation of either a TNFi or vedolizumab. Secondary outcomes included assessing the clinical usage patterns of advanced therapies in UP.

TNF inhibitors (infliximab-, adalimumab-, and golimumab-treated patients) were analyzed as a single class to preserve statistical power, and because these therapies share a common mechanism of action. The individual TNFi agents, particularly infliximab and adalimumab, may differ in pharmacokinetics, onset of action, and patterns of clinical use. To address this potential heterogeneity, we performed a prespecified sensitivity analysis comparing adalimumab with vedolizumab [10].

Steroid use was defined as use of prednisone, methylprednisolone, budesonide, hydrocortisone, prednisolone, beclomethasone or dexamethasone after starting the advanced therapy (Supplementary Table S2). Current Procedural Terminology (CPT) and ICD-10 codes for total abdominal colectomy, total proctocolectomy, proctectomy, total proctocolectomy with ileal pouch–anal anastomosis, colostomy and ileostomy were used to identify patients undergoing UC-related surgery during the follow-up (Supplementary Table S2). CPT codes for Hospital Inpatient and Observation Care services, urgent care visits and ER visits were used to assess the outcome of all-cause ER visits and hospitalizations. Infusion visits coded with CPT codes 96360–96379 were excluded to avoid counting infliximab or vedolizumab infusions as hospital encounters (Supplementary Table S2).

### 2.3. Statistical Methods

Statistical analyses were performed using TriNetX Analytics platform (TriNetX, Cambridge, MA, USA) and results were obtained on 23 January 2025. Propensity score matching (PSM) via TriNetX (R 4.0.2) was employed to adjust for confounders, including age, gender, ethnicity, nicotine use, obesity, pre-biologic hemoglobin, albumin, CRP and fecal calprotectin values, as well as baseline use of mesalamine, steroids and thiopurines. These variables were chosen prior to initiation of biologic therapy which was defined as the index event. Upon running the propensity score function, patients are matched based on propensity scores to create a 1:1 ratio between cohorts using nearest-neighbor (greedy) values without replacement. Cohorts are randomly shuffled prior to matching. Balance between cohorts post-matching was assessed using standardized mean differences (SMDs), with values < 0.1 indicating adequate balance. Categorical variables were compared using chi-square tests, and continuous variables were analyzed using two-tailed *t*-tests. Adjusted odds ratios (aORs) with 95% confidence intervals (CIs) were calculated for outcomes before and after matching.

## 3. Results

Among 641 patients with UP receiving advanced therapy across 140 HCOs, the most commonly used treatments were adalimumab (39%), infliximab (27%), and vedolizumab (26%). Ustekinumab was used in 12% of patients, while tofacitinib and upadacitinib were each used in 6%. Less frequently prescribed therapies included golimumab, risankizumab, guselkumab, ozanimod, and etrasimod (each 2%), as well as certolizumab (1%).

A total of 463 bio-naive patients were identified, of which 328 were treated with first-line TNFis and 135 with first-line vedolizumab. In the TNFi cohort (60% female; mean age 47.9 ± 17.4 years), adalimumab was more commonly used than infliximab (58.5% vs. 41.5%). Age, race, gender, nicotine dependence, and proportion of patients who were overweight or had obesity were comparable between patients on TNFis and vedolizumab without significant differences. Statistical differences were present prior to matching in fecal calprotectin (pre-PSM SMD: 0.14; post-PSM SMD: 0.07), serum hemoglobin (pre-PSM SMD: 0.30; post-PSM SMD: 0.03), serum albumin (pre-PSM SMD: 0.11; post-PSM SMD: 0.02) and in serum CRP (pre-PSM SMD: 0.23; post-PSM SMD: 0.12). Use of medications such as mesalamine, prednisone, hydrocortisone, immunomodulators and topical tacrolimus was also higher in the TNFi group compared to the vedolizumab group at the time of initiating biologic therapy (SMD > 0.1). PSM substantially improved covariate balance in all variables except for CRP where the SMD improved from 0.23 to 0.12 with higher CRP values in the TNFi group. (Table 1). Additionally, prior to starting advanced therapy, 3% of patients in the TNFi group and 8% in the vedolizumab group had received tacrolimus which was adequately matched after PSM.

In terms of outcomes, patients treated with TNFis had significantly lower odds of ER visits and/or hospitalizations compared to those on vedolizumab at both 6 months (16.8% vs. 26.4%; aOR 0.56, *p* = 0.02) and 12 months (24.2% vs. 39.7%; aOR 0.48, *p* = 0.01). There were no significant differences in the outcomes of steroid use or colectomy between TNFis and vedolizumab at 6, 12, and 24 months. (Table 2). At 24 months, the overall incidence of colectomy remained low in both groups.

PSM resulted in 132 matched patient pairs. In the TNFi group, 72 patients (56.2%) received adalimumab, 48 patients (37%) received infliximab, and a smaller proportion (7%) received golimumab. Following PSM, similarly to the unmatched cohorts, TNFi-treated patients had lower odds of ED visits and/or hospitalizations at 6 months (14.3% vs. 25%, aOR 0.50, *p* = 0.03) and 12 months (16.1% vs. 35.2%, aOR 0.35, *p* = 0.01). No significant differences in steroid use or colectomy rates were observed between the TNFi and vedolizumab groups at 6, 12, or 24 months after initiating therapy (Table 3). 

A sensitivity analysis of 132 matched patients comparing specifically adalimumab and vedolizumab showed a similarly reduced risk of ED visits and/or hospitalizations in the adalimumab group at both 6 months (16.6% vs. 28.7%; aOR 0.40, 95% CI 0.27–0.89, *p* = 0.02) and 12 months (19.6% vs. 30.3%; aOR 0.48, 95% CI 0.32–0.98, *p* = 0.04), consistent with the overall TNFi vs. vedolizumab findings (Table 4).

## 4. Discussion

Data on the long-term outcomes of first-line advanced therapies for UP remain limited, largely due to the exclusion of these patients from UC clinical trials [11]. Our study provides, for the first time, two-year comparative outcomes for refractory UP patients treated with TNFis or vedolizumab as first-line advanced therapy. Prior to matching, several laboratory and treatment-related variables suggested greater disease severity in the TNFi group, including higher fecal calprotectin and CRP levels, lower hemoglobin and albumin, and greater use of corticosteroids and immunomodulators at biologic initiation. These differences likely reflect treatment selection patterns in routine practice, where TNFis may be preferentially initiated in patients with more severe disease. Propensity score matching substantially improved balance across measured covariates, although a small residual imbalance in CRP persisted. Accordingly, residual confounding by disease severity cannot be fully excluded and should be considered when interpreting the results.

No statistically significant differences were observed in steroid use or IBD-related surgical outcomes at 6, 12, and 24 months. TNFi therapy was associated with a lower risk of all-cause ER visits and/or hospitalizations at 6 and 12 months, both before and after propensity score matching. One potential explanation for this early divergence may relate to differences in pharmacologic profiles. TNF inhibitors are known to achieve a relatively rapid clinical response, whereas vedolizumab, due to its gut-selective anti-integrin mechanism, may have a more gradual onset of effect [12]. However, this remains speculative in the context of a retrospective database study, and causal mechanisms cannot be established.

By 24 months, healthcare utilization rates were similar between groups, suggesting that both therapies may offer comparable longer-term disease control. Importantly, these findings should be interpreted with caution, as ER visits and hospitalizations represent nonspecific outcomes that may be influenced by factors beyond disease activity. Additionally, no meaningful differences were observed in more direct proxies of disease control, and colectomy events were rare, limiting statistical power for this outcome.

Overall, differences in healthcare utilization may reflect a combination of factors, including unmeasured disease severity, treatment selection patterns, and healthcare access, rather than pharmacologic effects alone.

Two prior RCTs, ACT 1 and ACT 2, demonstrated the efficacy of infliximab in inducing remission in patients with UC [13]. In both trials, 55% of participants had left-sided or distal colitis, but patients with UP were excluded. Similarly, prospective studies of other agents approved for UC, such as adalimumab, golimumab, and vedolizumab, also excluded patients with distal UC or UP [14,15,16]. Data on ustekinumab are limited as well, since the UNIFI trial excluded patients with disease limited to the rectum [17]. Evidence-based guidance on optimal positioning and comparative effectiveness of first-line advanced therapies for refractory UP remains limited.

Previous studies on advanced therapies in UP have suggested favorable results for vedolizumab though this must be interpreted with caution as they were not based on head-to-head comparisons. In a study by Dubois et al. [1] among 36 patients with refractory UP, TNFi agents were the most commonly used first-line therapy, followed by vedolizumab. Treatment success defined as a clinical response according to the treating physician was achieved in 50% of TNFi-treated patients (with a 21-month follow-up) and in 67% of those treated with vedolizumab (11-month follow-up). A multicenter retrospective cohort study from Belgium involving 167 consecutive UP patients treated with advanced therapies found that steroid-free clinical remission in the short term was more frequently achieved in biologic-naive patients treated with vedolizumab. Additionally, drug persistence was higher in patients who received vedolizumab [18]. None of these were direct comparisons of both these medications. A recent retrospective cohort study comparing both directly reported higher steroid-free clinical remission at 12 months in patients receiving first-line vedolizumab compared with TNFis for UP [5]. That study included small sample sizes (34 vedolizumab and 32 TNFi patients). Our larger sample size and the PSM used in our study design may explain the differing results of lower odds of ER visits and/or hospitalizations with TNFi and comparable steroid use.

TNFis have been independently studied in UP patients. In a French study, 104 consecutive patients with refractory UP on TNFis reported clinical remission in 50% and mucosal healing in 60% at 24 months [4]. Similar remission rates in refractory UP on infliximab were observed in a study done by Bouguen et al. in 420 patients showing remission in 69% of patients and 15% were primary non-responders to infliximab [9]. Similarly, in our study the approximate complication rate of steroid use or all-cause hospitalizations ranged from 16 to 25% in the TNFi group over a span of 2 years.

A systematic review of therapies for refractory UP in 2022 concluded that TNFis have shown efficacy in treating UP but expose patients to the risk of systemic adverse events [19]. Gut-selective biologic therapies, such as vedolizumab, have favorable safety profiles and may be preferred for treating refractory UP, particularly in patients without extraintestinal manifestations [5,20].

UP is associated with a very low colectomy rate at 1, 2, and 5 years post-diagnosis, likely due to its limited colonic involvement and the morbidity of an abdominal colectomy [2]. Patients with isolated proctitis rarely require colectomy for refractory disease. Their generally more indolent disease course has also contributed to their frequent exclusion from clinical trials and the resulting scarcity of robust evidence regarding treatment efficacy in this population. Similarly, our findings showed a low overall surgery rate in the cohort (<10 cases), consistent with prior studies reporting lower colectomy rates in UP compared to pancolitis [21]. Because colectomy events were infrequent (<10 cases), the study was underpowered to support a meaningful comparative analysis of this outcome. Colectomy is an exceptionally uncommon outcome in isolated UP and its low event rate in our study aligns with the prior studies of this distinct disease phenotype [21].

This study’s strengths include its large, real-world sample of UP patients treated with first-line TNFis or vedolizumab with use of the TriNetX Global Collaborative Network that includes data from multiple healthcare organizations globally, enhancing generalizability of the results. The database is prospectively maintained, and propensity score matching was used to reduce measured confounding. Outcomes were analyzed over 6, 12, and 24 months, extending insights beyond induction. To our knowledge, this is the largest real-world study comparing TNFi and vedolizumab outcomes in UP. An additional strength of our study is the ability to characterize real-world biologic utilization patterns in patients with UP. Specifically, we were able to describe the relative distribution of biologic therapies used in this population, an area that is not well characterized in the existing literature. These findings provide complementary context to the comparative analyses by reflecting on prescribing patterns in routine clinical practice.

This study has several limitations. First, the absence of endoscopic confirmation that disease was confined to the rectum represents an important limitation. The use of the TriNetX database relies on ICD-10 and CPT administrative codes for ulcerative proctitis, which may introduce misclassification bias. We attempted to mitigate this by excluding patients with diagnostic codes for left-sided colitis, ulcerative proctosigmoiditis, pancolitis, extensive ulcerative colitis, or Crohn’s disease throughout the study period. Patients with any prior or subsequent coding for more extensive colitis were excluded to improve cohort specificity.

Additionally, the lack of data on disease duration, endoscopic severity, histologic activity, indication for biologic selection, and independent confirmation of disease extent represents a limitation inherent to database studies. To partially account for disease severity, we incorporated available markers of disease severity, including CRP, fecal calprotectin, and baseline use of corticosteroids and immunomodulators prior to biologic initiation. As with most database studies, some covariate data were missing; however, the large sample size and global representation help mitigate this limitation. Given the retrospective nature of the TriNetX database, capturing specific low-grade adverse events is a known limitation. Additionally, we could not distinguish between topical and oral steroid use in the outcomes following treatment initiation. For example, the RxNorm code for budesonide could refer to either budesonide foam or oral budesonide. Despite this, we consider the need for any form of steroids, whether topical or oral, after starting a biologic such as TNFis or vedolizumab to indicate inadequate disease control and therefore a complication. The exact indication for biologic selection cannot be determined from database studies, as clinical notes are not available for review. We observed a trend toward greater use of TNFis in patients with more severe laboratory markers of inflammation, which may provide insight into treatment selection patterns; however, this remains speculative and cannot establish causality given the limitations of the dataset.

Additionally, due to the retrospective design, calendar-time effects may have influenced both treatment allocation and outcome rates independent of comparative drug effectiveness.

To conclude, in this propensity-matched analysis, no statistically significant differences were observed between TNFis and vedolizumab in corticosteroid use over two years. Colectomy events were infrequent, as expected in patients with isolated UP, limiting meaningful comparison of this rare outcome. TNFi use was associated with lower rates of ER visits and/or hospitalizations at 6 and 12 months, reflecting lower short-term healthcare utilization; however, these measures may not directly capture disease activity. These findings should be interpreted in the context of potential residual confounding. Overall, these findings suggest comparable corticosteroid utilization between TNFis and vedolizumab as first-line biologic therapy for isolated UP while lower healthcare utilization observed with TNFis warrants further prospective investigation to better define potential differences.

## Figures and Tables

**Table 1:** Baseline characteristics of patients in the TNFi and vedolizumab groups before initiation of the biologic, before and after propensity score matching.

Variables	Before Propensity Score Matching				After Propensity Score Matching			
	TNFis (N = 328) N (%)	Vedolizumab (N = 135) N (%)	*p*-Value	SMD *	TNFis (N = 132) N (%)	Vedolizumab (N = 132) N (%)	*p*-Value	SMD *
Demographics								
Age at time of inclusion (years), mean ± SD	47.9 ± 17.4	46.5 ± 17	0.44	0.07	47.4 ± 17.4	46.3 ± 16.7	0.58	0.07
Female gender, n (%)	196 (60)	76 (56.2)	0.46	0.07	67 (50.7)	76 (57.5)	0.26	0.03
Ethnicity, n (%)								
White	225 (68.8)	91 (67.4)	0.76	0.03	90 (68.1)	89 (67.4)	0.89	0.01
African American	23 (7)	10 (7.4)	0.88	0.01	10 (7.5)	10 (7.5)	1.0	<0.01
Hispanic	13 (3.9)	10 (7.4)	0.12	0.14	10 (7.5)	10 (7.5)	1.0	<0.01
Nicotine Dependence, n (%)	20 (6.3)	10 (7.4)	0.5	0.07	10 (7.5)	10 (7.5)	1.0	<0.01
Overweight and Obesity, n (%)	19 (5.8)	10 (7.4)	0.51	0.06	10 (7.5)	10 (7.5)	1.0	<0.01
Laboratory Values (g/dL), mean ± SD ^a^								
Hemoglobin	12.8 ± 2.2	13.4 ± 1.71	0.06	0.30	12.8 ± 2.38	13.3 ± 1.73	0.17	0.03
Albumin	3.9 ± 0.7	4.2 ± 0.6	0.27	0.11	4.02 ± 0.7	4.2 ± 0.6	0.13	0.02
Fecal calprotectin	543 ± 693	488 ± 781	0.86	0.14	442 ± 1146	427 ± 551	0.40	0.07
CRP	19 ± 42.5	12.9 ± 24.1	0.48	0.23	17.9 ± 32.4	10.4 ± 20.7	0.18	0.12
Medication use at the time of initiating biologic therapy, n (%) ^b,c^								
Mesalamine	169 (53.8)	65 (49.2)	0.21	0.12	52 (42)	51(40.4)	0.79	0.03
Prednisone	102 (32)	31 (23.8)	0.07	0.19	32 (25)	30 (23.8)	0.76	0.04
Hydrocortisone	68 (21.3)	18 (13.6)	0.06	0.2	17 (13.4)	17 (13.4)	1.0	<0.01
Tacrolimus	10 (3.0%)	11 (8.0%)	0.21	0.13	4 (3.0%)	4 (3.0%)	1.0	<0.01
Azathioprine	40 (12.5)	11 (8.3)	0.19	0.13	11 (8.7)	10 (7.5)	0.8	0.02
6-Mercaptopurine	22 (7)	10 (7.4)	0.08	0.16	10 (7.5)	10 (7.5)	1.0	<0.01

* SMD, standardized mean difference; TNFis, tumor necrosis factor inhibitors. ^a^ Laboratory results within 1 year of index event of starting biologic therapy. ^b^ Medication use within 1 year prior to initiating biologic therapy. ^c^ Patients could have been on either of these medications or a combination of these medications.

**Table 2:** Comparison of outcomes between TNFi and vedolizumab groups at 6 months, 12 months and 24 months before propensity score matching.

Outcome	TNFis (%)	Vedolizumab Cohort (%)	Odds Ratio ^#^	95% CI	*p*-Value
6 months					
Oral and/or intravenous steroid use	24.0	20.5	1.22	0.79–1.70	0.42
Total proctocolectomy or proctectomy	N < 10	N < 10	NA	NA	NA
Hospitalization or ER visits	16.8	26.4	0.56	0.34–0.90	**0.02**
12 months					
Oral and/or intravenous steroid use	27.6	22.0	1.34	0.84–2.16	0.21
Total proctocolectomy or proctectomy	N < 10	0	NA	NA	NA
Hospitalization or ER visits	24.2	39.7	0.48	0.27–0.85	**0.01**
24 months					
Oral and/or intravenous steroid use	31.5	24.2	1.4	0.91–2.26	0.11
Total proctocolectomy or proctectomy	N < 10	0	NA	NA	NA
Hospitalization or ER visits	25.5	29.4	0.82	0.52–1.28	0.38

^#^ Vedolizumab cohort is the reference group. Abbreviations: NA, not applicable; N, number; TNFis, tumor necrosis factor inhibitors; ER, emergency room; CI, confidence interval.

**Table 3:** Comparison of outcomes between TNFi and vedolizumab groups at 6 months, 12 months and 24 months after propensity score matching.

Outcome	TNFis (%)	Vedolizumab (%)	Odds Ratio ^#^	95% CI	*p*-Value
6 months					
Oral and/or intravenous steroid use	17.4	21.2	0.7	0.42–1.44	0.43
Total proctocolectomy or proctectomy	0	N < 10	NA	NA	NA
Hospitalization or ER visits	14.3	25	0.50	0.27–0.94	**0.03**
12 months					
Oral and/or intravenous steroid use	21.2	22.2	0.90	0.51–1.64	0.76
Total proctocolectomy or proctectomy	0	0	NA	NA	NA
Hospitalization or ER visits	16.1	35.2	0.35	0.15–0.8	**0.01**
24 months					
Oral and/or intravenous steroid use	22.7	25.1	0.88	0.50–1.55	0.66
Total proctocolectomy or proctectomy	N < 10	0	NA	NA	NA
Hospitalization or ER visits	25.7	28.1	0.89	0.51–1.53	0.67

^#^ Vedolizumab cohort is the reference group. Abbreviations: NA, not applicable; N, number; TNFis, tumor necrosis factor inhibitors; ER, emergency room; CI, confidence interval.

**Table 4:** Sensitivity analysis between adalimumab and vedolizumab after PSM.

Outcome	Adalimumab N = 132 (%)	Vedolizumab N = 132 (%)	Odds Ratio ^#^	95% CI	*p*-Value
6 months					
Oral and/or intravenous steroid use	23.5	25	0.9	0.52–1.6	0.77
Total proctocolectomy or proctectomy	0	N < 10	NA	NA	NA
Hospitalizations or ER visits	16.6	28.7	0.4	0.27–0.89	**0.02**
12 months					
Oral and/or intravenous steroid use	26.5	26.5	1	0.58–1.72	1
Total proctocolectomy or proctectomy	0	N < 10	NA	NA	NA
Hospitalizations or ER visits	19.6	30.3	0.56	0.32–0.99	**0.04**
24 months					
Oral and/or intravenous steroid use	28.0	28.7	0.96	0.56–1.64	0.89
Total proctocolectomy or proctectomy	N < 10	0	NA	NA	NA
Hospitalization or ER visits	25	30.3	0.82	0.44–1.31	0.33

^#^ Vedolizumab cohort is the reference group. Abbreviations: NA, not applicable; N, number; ER, emergency room; CI, confidence interval.

## Data Availability

Data available in a publicly accessible repository. The data presented in this study are openly available in TriNetX database website.

## Supplementary Materials

The following supporting information can be downloaded at: https://www.mdpi.com/article/10.3390/biomedicines14051135/s1, Table S1: List of International Classification of Diseases (ICD-10) codes used in this study. Table S2: RxNorm and CPT codes used for outcomes analysis.
