# Supplementary material for: Long-Term Comparative Outcomes of TNF-α Antagonists vs. Vedolizumab as First-Line Biologic Therapy for Refractory Ulcerative Proctitis: A Propensity-Matched Study
Source: Biomedicines. 2026 May 17;14(5):1135. doi: 10.3390/biomedicines14051135 (PMC13204673; doi:10.3390/biomedicines14051135)
Supplement: Supplementary file 1 [file biomedicines-14-01135-s001.zip › biomedicines-4242859-supplementary.pdf]

Supplemental Content

Patient Selection

Patients with isolated ulcerative proctitis were identified using ICD codes as specified in the table below. For identifying patients using anti-TNF agents as first line, RxNorm codes of Infliximab, Adalimumab, Golimumab and Certolizumab were used to further identify the group of UP patients on these therapies and patients with other autoimmune conditions, ulcerative pancolitis, left-sided colitis, Crohn's disease, and those with vedolizumab, ustekinumab, rizankizumab, mirikizumab, ozanimod, etrasimod, tofacitinib and upadacitinib were excluded. Similarly, the comparison group identified patients with ulcerative proctitis on vedolizumab therapy and excluded any patients with diagnoses of autoimmune conditions, ulcerative pancolitis, left-sided colitis, Crohn's disease, and those on infliximab, adalimumab, golimumab, certolizumab, ustekinumab, rizankizumab, mirikizumab, ozanimod, etrasimod, tofacitinib and upadacitinib were excluded.

Supplemental Table S1: List of International Classification of Diseases (ICD-10) codes used in this study.

**Inclusion Diagnoses**	ICD 10 codes
Ulcerative Proctitis	K51.2 Ulcerative (chronic) proctitis K51.20 Ulcerative proctitis without complications<br>K51.21 Ulcerative (chronic) proctitis with complications<br>K51.211 Ulcerative (chronic) proctitis with rectal bleeding<br>K51.212 Ulcerative (chronic) proctitis with intestinal obstruction<br>K51.213 Ulcerative (chronic) proctitis with fistula<br>K51.214 Ulcerative (chronic) proctitis with abscess<br>K51.218 Ulcerative (chronic) proctitis with other complication<br>K51.219 Ulcerative (chronic) proctitis with unspecified complications
**Exclusion Diagnoses**	
Ulcerative Pancolitis	K51.0 Ulcerative (chronic) pancolitis<br>K51.00 Ulcerative (chronic) pancolitis without complications

	K51.01 Ulcerative (chronic) pancolitis with complications<br>K51.011 Ulcerative (chronic) pancolitis with rectal bleeding<br>K51.012 Ulcerative (chronic) pancolitis with intestinal obstruction<br>K51.013 Ulcerative (chronic) pancolitis with fistula<br>K51.014 Ulcerative (chronic) pancolitis with abscess<br>K51.018 Ulcerative (chronic) pancolitis with other complication<br>K51.019 Ulcerative (chronic) pancolitis with unspecified complications
Ulcerative Left-sided colitis	K51.5 Left sided colitis<br>K51.50 Left sided colitis without complications<br>K51.51 Left sided colitis with complications<br>K51.511 Left sided colitis with rectal bleeding<br>K51.512 Left sided colitis with intestinal obstruction<br>K51.513 Left sided colitis with fistula<br>K51.514 Left sided colitis with abscess<br>K51.518 Left sided colitis with other complication<br>K51.519 Left sided colitis with unspecified complications
Other specified noninfective gastroenteritis and colitis	K52.9
Indeterminate colitis	K52.3
Crohn’s disease	K50.0-50.9
Psoriasis	L40
Rheumatoid Arthritis	M05.x, M06.x
Systemic Lupus Erythematosus	M32
Ankylosing spondylitis	M45.x
Multiple sclerosis	G35
Vasculitis/Unspecified Arteritis	I77.6

Supplemental Table S2: RxNorm and CPT codes used for outcomes analysis

Steroid use	Prednisone, Methylprednisolone, Budesonide, Hydrocortisone, Dexamethasone, Prednisolone, Beclomethasone	RxNorm: 8640, 6902, 19831, 5492, 3264, 8638, 1347
Colectomy	Total abdominal colectomy	CPT 44141, 44143, 44144, 44146, 44147, 44150, 44151, 44206, 44208, 44210
	Total proctocolectomy	CPT 44155, 44156, 44212
	Colostomy or ileostomy status	ICD 10 Z93.3, Z43.3, K94.0, K94.01-03. Z93.2
	Proctectomy	CPT 45110, 45111, 45113, 45114, 45116, 45119, 45120, 45123
	Total proctocolectomy with IPAA	CPT 44152, 44153, 44157, 44158, 44211, 45113
All-cause hospitalization, urgent care or emergency care visits	CPT 99252-99255, 99218-99236, 99281-99284, 1013711, 1013699, 1013659, 1013668, 1013682, 1013675, 1013659, 1013729, 1013648	
